# Cancer Stem Cell Relationship with Pro-Tumoral Inflammatory Microenvironment

**DOI:** 10.3390/biomedicines11010189

**Published:** 2023-01-11

**Authors:** Ferenc Sipos, Györgyi Műzes

**Affiliations:** Immunology Division, Department of Internal Medicine and Hematology, Semmelweis University, 1085 Budapest, Hungary

**Keywords:** cancer stem cells, inflammation, tumor microenvironment, leukocyte reprogramming, tumor-associated macrophages, cancer-associated fibroblasts, regulatory T cells

## Abstract

Inflammatory processes and cancer stem cells (CSCs) are increasingly recognized as factors in the development of tumors. Emerging evidence indicates that CSCs are associated with cancer properties such as metastasis, treatment resistance, and disease recurrence. However, the precise interaction between CSCs and the immune microenvironment remains unexplored. Although evasion of the immune system by CSCs has been extensively studied, new research demonstrates that CSCs can also control and even profit from the immune response. This review provides an overview of the reciprocal interplay between CSCs and tumor-infiltrating immune cells, collecting pertinent data about how CSCs stimulate leukocyte reprogramming, resulting in pro-tumor immune cells that promote metastasis, chemoresistance, tumorigenicity, and even a rise in the number of CSCs. Tumor-associated macrophages, neutrophils, Th17 and regulatory T cells, mesenchymal stem cells, and cancer-associated fibroblasts, as well as the signaling pathways involved in these pro-tumor activities, are among the immune cells studied. Although cytotoxic leukocytes have the potential to eliminate CSCs, immune evasion mechanisms in CSCs and their clinical implications are also known. We intended to compile experimental findings that provide direct evidence of interactions between CSCs and the immune system and CSCs and the inflammatory milieu. In addition, we aimed to summarize key concepts in order to comprehend the cross-talk between CSCs and the tumor microenvironment as a crucial process for the effective design of anti-CSC therapies.

## 1. Introduction

The presence of cancer stem cells (CSCs) has been confirmed in several tumors. They play a fundamental role in tumor development, progression, metastasis, and relapse. Through their self-renewal capacity and differentiation potential, CSCs are able to maintain tumor heterogeneity. In addition, they are able to activate resistance mechanisms (e.g., altering the expression of drug export systems, reducing cell division, promoting epithelial-to-mesenchymal transition (EMT), increasing resistance to hypoxia through angiogenesis, being able to induce immune escape by reducing tumor antigens, and influencing the composition of cytokines and growth factors that determine the inflammatory environment) [1,2].

Identification of CSCs is not a simple task, as the boundary between tumor cells and CSCs is often unclear. In recent years, a number of CSC markers have been identified that can help define the CSC phenotype (Table 1) [3,4,5]. However, these markers are expressed not only by CSCs but also by embryonic or adult stem cells, and they are rarely expressed on normal tissue [6].

In healthy tissues, stem cells are located in a defined compartment. The interrelationship between stem cells and their environment is essential for the maintenance of the stem cell phenotype. CSCs, like conventional stem cells, are located in a specialized environment. Chronic inflammation has been recognized as a hallmark of cancer since the seminal publications of Colotta [7], Hanahan, and Weinberg [8,9]. CSCs can influence their own capabilities to enable their own survival and progression, as well as their interaction with their inflammatory environment [10]. In this review article, we aim to provide a concise overview of the recent findings regarding the reciprocal effects between chronic inflammation and cancer stem cells and how they promote the maintenance of the stem cell phenotype and thus disease progression. We aim to bring together specific experimental results that provide direct evidence for interactions between CSCs and the immune system and between CSCs and the inflammatory microenvironment.

## 2. CSCs Influence Their Own Capabilities by Different Mechanisms

In the past, cancer was described as a heterogeneous mass of cells composed of distinct subgroups, and numerous tumor models have attempted to explain the underlying cellular heterogeneity. The hierarchical model of CSCs posits the existence of clusters of malignant cells with varying proliferative and differentiative capacities, with the highest hierarchical level and the highest oncogenic capacity being associated with stem cell properties and successive differentiation potentials [11]. In accordance with the dynamic CSC concept, a feedback control system can be established between CSCs and tumor progenitor cells, indicating that cancer progenitor cells can acquire stem properties in response to certain microenvironmental signals [12,13]. In this context, it is becoming evident that inflammatory circumstances collaborate to induce deregulations, mutations, cell fusion, and other phenomena, ultimately resulting in conditions that promote CSC.

In the early stages of cancer development, the cancer cells are under intense immunological attack, so that the immune system destroys them. However, during this process, cells that are less immunogenic and therefore almost invisible to the immune system may be selected. Such cells include CSCs. CSCs are able to modify their own properties to ensure their survival. One possible way for CSCs to survive is to exit the cell cycle and enter a dormant state. This dormant state is regulated by Nanog Homeobox (NANOG) through wingless-related integration site (Wnt)/β-catenin signaling [14]. Compared to tumor mass cells, CSCs are able to ensure their own survival by enhancing the deoxyribonucleic acid (DNA) repair mechanisms [15], reducing apoptosis [16], and increasing the expression of certain drug efflux pumps (e.g., multi-drug resistance 1 /MDR1/, ATP binding cassette subfamily G member 2 /ABCG2/) [17].

**Figure 1:**
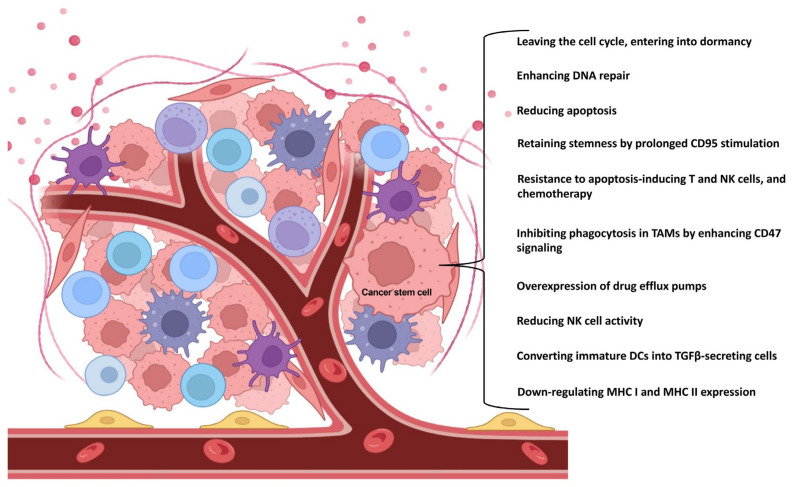
CSCs have multiple ways of ensuring their own survival. In addition to affecting basic cellular functions, they can also alter the anti-tumor function of the immune system. The figure was partly created with BioRender.com.

To further ensure their survival, CSCs can also evade the innate immune system in several ways [18]. Crucially, in glioblastoma, melanoma, and colorectal cancer, they are able to downregulate the expression of major histocompatibility complex (MHC) I and II molecules [19,20,21]. They are also able to convert a subset of immature dendritic cells (DCs) into transforming growth factor (TGF)β-secreting cells, which ultimately leads to the expansion of regulatory T cells (Tregs) in lymphoid organs [22]. They are also capable of reducing the activity of natural killer (NK) cells in several ways, e.g., by decreasing the expression of natural killer group 2, member D (NKG2D) ligands in glioblastoma and breast cancer [19,23]; by decreasing the expression of ligands for NK cell activating receptors such as NKp44, NKp30, NKp46, and CD16 [24,25]; or by increasing the expression of NK cell inhibitory receptor ligands [24,25]. In lung, pancreatic, and hepatocellular cancers, they are also able to inhibit their own phagocytosis by enhancing CD47 (“don’t hurt me”) signaling via SIRPα on tumor-associated macrophages (TAMs) [26,27,28,29]. In addition, they are resistant to apoptosis-inducing T and NK cells and chemotherapy. According to the results from prostate cancer, this is mainly achieved by increasing the expression of CD200, CD95/FasL, B-cell lymphoma 2 (Bcl2), B-cell lymphoma-extra large (Bcl-xL), and survivin [30,31]. Survivor CSCs are also capable of retaining their stemness. Through prolonged CD95/Fas stimulation, they promote signal transducer and activator of transcription (STAT)1 activation by type I interferons (IFNs) [32], which in turn enhances the expression of stem-like markers (Figure 1) [33].

Despite the promising results, CSCs’ biology and immunomodulatory ability are not yet entirely understood. Understanding the mechanisms that guide the plasticity and phenotype of CSCs, such as immunogenicity, proliferative activity, differentiation, or migration during tumoral development, as well as the recognition of CSC-specific markers, are the main drawbacks for CSC-targeted anti-cancer therapies, which aim to eradicate the tumor completely and effectively prevent relapses.

## 3. The Mutual Role of TME and CSCs in Immunomodulation and Stem Cell Niche Maintenance

The heterogeneous (i.e., differences in immune cell infiltration and the amount of necrotic tumor cells, interstitial pressure, genetic and epigenetic alterations) and location dependent (i.e., tumor periphery vs. tumor core) tumor microenvironment (TME) is consisting of stroma, extracellular matrix, vasculature, immune cells, and different signaling molecules and pathways (i.e., Notch-, Wnt-, and Hedgehog-pathways) [34,35]. Crosstalk between CSCs and cells in the TME is variable and extensive, involving interconnections between CSCs, tumor stromal cells, and non-CSCs. It is assumed that CSCs inhabit a particular sub-compartment of TME known as the CSC niche. A favorable microenvironment and the absence of specific stimuli that affect cell proliferation keep CSCs quiescent [36]. CSCs survive tumor eradication in quiescence but do not lose their malignant potential, orchestrating the transition to the escape phase. According to acute leukemia studies, repeated tumor growth is triggered by the aggressive and slowly dividing CSC clone [37]. During the escape phase, CSCs secrete cytokines, chemokines, and soluble factors to blunt and alter immune functions and develop immune tolerance in order to create a pro-tumor niche [38]. Tregs, TAMs, and myeloid-derived suppressor cells (MDSCs) are the main organizers of this process, as they mainly enhance the formation of an immune-tolerant TME by secreting interleukin (IL)10, TGFβ, and prostaglandins, as found in colorectal cancer (CRC) [39,40,41,42]. They also inhibit the secretion of IL12 by DCs, block the efficient Th1 response, and inhibit NK, natural killer T (NKT), and effector T cells [40,41,42,43]. During the further development of TME, the formation of angiogenesis-promoting N2-polarized tumor-associated neutrophil granulocytes (TANs) is enhanced through immunosuppressive factors and cytokines (e.g., TGFβ) [44].

In cancer patients, a so-called emergency myelopoiesis is observed, whereby TAMs and MDSCs proliferate in abundance, leading to an abnormal overgrowth of tumor-supporting myeloid cells [45,46]. In addition to the local immune cell dysregulation that occurs in TME, cancers also alter the differentiation of bone marrow progenitors through systemic effects, thereby affecting the extent, composition, and specific functions of hemopoiesis [47,48]. Myeloid cells that have been transferred from the bone marrow to the periphery are transported to the tumor, where they encounter extreme conditions (e.g., hypoxia, low pH, low glucose, and inflammatory signals). The altered microenvironment further enhances their reprogramming towards a pro-tumor phenotype [43,49]. CSCs promote the differentiation of immature myeloid cells by secreting inflammatory mediators (e.g., IL10, IL13) [50]. In addition, granulocyte colony-stimulating factor (G-CSF), C-C motif chemokine ligand (CCL)2, CCL15, and chemokine (C-X-C motif) ligand (CXCL)12 produced by CSCs recruit additional MDSCs to the TME in colon cancers [51,52]. Experimental results in pancreatic cancer have demonstrated that monocyte-derived MDSCs (M-MDSCs) promote CSC expansion and the expression of genes related to the epithelial-to-mesenchymal transition [53]. Similarly, in CRC, granulocyte-derived MDSCs (G-MDSCs) promote CSC formation via exosomes, especially within hypoxic microenvironments [54].

In addition, in uterus, breast, and hepatocellular cancers MDSCs can promote the emergence and maintenance of the CSC phenotype in several ways (e.g., C-terminal binding protein 2 /CtBP2/ inhibition by micro-ribonucleic acid (miRNA)10 1; increased prostaglandin E2 (PGE2) production; increased IL6 and nitric oxide (NO) production by involving the STAT3 signaling) [55,56,57,58]. TAMs also increase the number of CSCs through the induction of the STAT3/IL6 pathway in liver cancer [57] and promote the self-renewal, tumorigenicity, chemoresistance, and migration of CSCs via interferon-stimulated gene 15 (ISG15) secretion in pancreas and nasopharyngeal cancers [59,60]. M2-polarized TAMs promote CSC proliferation and invasion in liver cancer through the secretion of TGFβ, platelet-derived growth factor (PDGF), CXCL12, and IL8 [61,62]. They also stimulate angiogenesis and maintain stem cell properties throughout VEGF production in breast cancer [63,64]. This suggests that TAMs promote tumor progression by supporting the CSC niche [58,65].

Tregs are also an important component of the TME. Tregs are essentially immunosuppressive and act against tissue damage caused by inflammation [66]. In tumors, however, Tregs suppress the anti-tumor effect of tumor-infiltrating immune cells, thereby promoting tumor escape. The functions of Tregs and their polarization between “anti-tumor” and “pro-tumor” states are regulated by complex molecular and cellular interactions. The binding of semaphorin-4a (Sema-4a) expressed on immune cells to neuropilin-1 (Nrp-1), a receptor for Tregs, enhances the survival and immunosuppressive activity of Tregs. The Nrp-1/Sema-4a pathway is absolutely required for the protection and prolongation of Treg survival in TME [66,67]. Other T cell types can interconvert between phenotypes as well. IL17 producing CD4^+^ Th17 and Th2 cell are able to switch to IFNγ producing ones via epigenetic, metabolic, and cytokine signaling pathways [68]. Furthermore, in non-small cell lung cancer (NSCLC), memory (CD4^+^ forkhead box P3/Foxp3/^+^ CD25^high^ CD27^+^ CD45RA^−^) Tregs can also be transformed into Th17-like phenotype, expressing C-C Motif Chemokine Receptor (CCR)6 and IL17 [69,70].

Mesenchymal stem cells (MSCs) can promote the chemoresistance of CSCs both directly and indirectly. In breast, colon, and gastric cancers, as well as in glioma, and acute lymphoid leukemia, they contribute to CSC survival during various anti-cancer treatments by secreting fatty acids, exosomes, chemoattractants and growth factors, and by cell–cell contact via miRNA upregulation [71,72,73,74,75,76,77,78,79]. Based on the results in gastric cancer, lung, liver, and ovarian cancers, cancer-associated fibroblasts (CAFs) derived from MSCs, fibroblasts, or epithelial cells also promote EMT and the survival of the CSCs’ stem cell phenotype throughout paracrine actions (IL6, IL1β, and CXCL12 secretion) and via insulin-like growth factor 1 receptor (IGF1R), TGFβ, STAT1, and nuclear factor-κB (NF-kB) pathways [80,81,82,83,84,85].

Besides the positive effects of TME on CSCs, activated CSCs provide favorable conditions for the M2 polarization of TAMs and their pro-tumorigenic effect as well [40]. Several different factors in gliomas, glioblastomas, and ovarian cancers may play a role in this process, such as periostin (an extracellular matrix protein), colony stimulating factors, CCL2, cyclooxygenase 2 (COX2), or TGFβ [86,87,88,89,90]. CSCs contribute to the development of their own vascular network through vascular endothelial growth factor (VEGF) production [63,64], and promote their own stem cell development through the activation of Wnt-signaling via the interaction between CSCs and TAMs [91].

In addition to the action of pro-tumorigenic cytokines via the paracrine pathway, the CSC-derived secretome also plays an important role in the establishment and maintenance of TME. Glioblastoma CSC-derived exosomes can stimulate M2 polarization, programmed death-ligand 1 (PD-L1) expression, and the production of monocyte chemotactic protein 3 (MCP-3) and CXCL1, which promote myeloid cell recruitment, through the STAT3 pathway in glioblastoma [92]. Using the same secretome in glioma, circulating monocytes produce increased IL10 and arginase 1 (Arg1), decrease Human Leukocyte Antigen DR isotype (HLA-DR) expression, and thereby transform into M-MDSC-like cells [93]. In breast cancer, exosomes containing TGFβ, complement component 1q (C1q), and semaphorins also promote M2-directed (immunosuppressive) polarization and differentiation of the M-MDSCs [94]. Maturation of DCs and T cell responses can be inhibited by HLA-G-containing extracellular vesicles, which favor renal tumor cell immune escape mechanisms [95]. Exosomes of CRC-derived CSCs increase neutrophil granulocyte lifespan and promote the formation of pro-tumorigenic phenotype TANs by increasing the IL1β expression [96]. Melanoma CSCs can educate neutrophils to support cancer progression in several ways, such as neutrophil recruitment via TGF-β, IL6, and IL8, enhancing N2-polarization by the activation of ERK, STAT3, and P38 pathways, as well as the overexpression of CXCR2 and NF-kB [97]. MSC-derived secretome of TME also favors the tumorigenic inflammatory response of TAMs by decreasing pro-inflammatory and increasing anti-inflammatory cytokine production [98]. According to the results from liver, gastric, renal, and thyroid cancers, exosomes derived from CSCs can influence apoptosis, angiogenesis, EMT, and metastasis formation by modulating the expression of p53, Bcl2, VEGF, angiopoietin1, TGFβ, matrix metalloproteinase (MMP)2 and MMP9, as well as by displaying a pro-tumor miRNA profile [99,100,101,102,103].

Along with the paracrine effects of the cytokines and the secretome-mediated TME formation possibilities, TAMs interact with CSCs through direct cell–cell contact. The binding of CD90/Thy-1 of TAMs and ephrin-A receptor 4 (EphA4) expressed on the surface of breast CSCs induces IL6, IL18, and granulocyte-macrophage colony-stimulating factor (GM-CSF) production, which promote the maintenance of a stem cell-like microenvironment [104].

**Figure 2:**
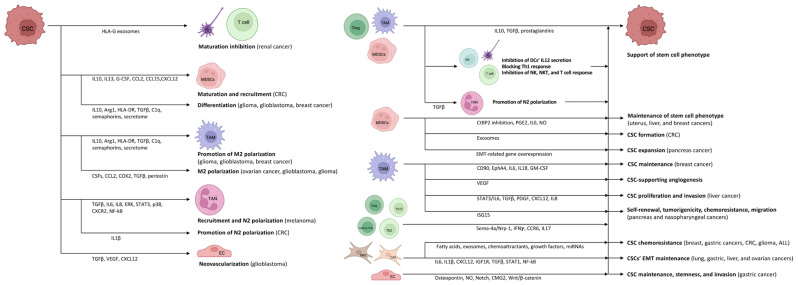
There is an intense association between CSCs and the inflammatory TME. CSCs are able to use immune-competent cells to their own advantage. At the same time, inflammatory TME cells promote the survival of CSCs. Green arrows indicate a stimulatory effect, while the red arrow indicates inhibition. The figure was partly created with BioRender.com.

CSCs may exhibit potent angiogenic properties and contribute to the recruitment of blood vessels during cancer. Vascular endothelial cells (ECs) provide CSCs with supportive signals through cell-to-cell interactions [105]. Under the impact of TGFβ, glioblastoma CSCs are able to generate pericytes that enable neovascularization and cancer progression [106]. CSCs produce the angiogenic molecules VEGF and CXCL12 to stimulate EC angiogenesis. ECs, in turn, secrete stemness-maintaining substances such as NO and osteopontin, and stimulate Notch signaling [107]. Anti-VEGF medication that inhibits ECs can, surprisingly, also be tumorigenic. The anti-VEGF medication may create hypoxia within the TME, which unexpectedly induces VEGF within the TME via a negative feedback loop [108]. This hypoxic environment can also inhibit CSC differentiation, increase cell treatment resistance, and boost stem-like characteristics in non-CSCs [108,109]. Moreover, ECs upregulate capillary morphogenesis gene 2 (CMG2) protein to increase stemness, invasion, and metastasis of CSCs via activating the Wnt/β-catenin pathway observed in gastric cancer [110] (Figure 2).

Future new therapeutic techniques will require a comprehensive understanding of the relationship between TME and CSCs. In this direction, researchers have previously identified CSCs as the major source of cancer relapse and chemotherapeutic drug resistance in numerous solid tumors. In addition to this cell subpopulation, TAMs, TANs, CAFs, T cell subsets, and other immune cells, as well as their secretome, participate in interactions that can benefit or hinder the fight against cancer. As a result, targeting the cellular or secretome components of the TME provides a potential cancer therapeutic approach.

## 4. The CSC-TME Crosstalk in Highly Inflammatory Cancers

The link between inflammation and the development of cancer is rather complex [111]. In the case of acute inflammation following tumor antigen uptake or activation by a Toll-like receptor (TLR) agonist, mature DCs may regulate the anti-tumor immune response by modulating the inflammatory response through various mechanisms (e.g., cross-presentation of tumor antigens and priming of tumor-specific CD8^+^ T cells, polarization of immune cells towards the anti-tumor phenotype, recruitment of NK cells, thereby maintaining the T cell response) [111,112]. If the acute inflammation is not resolved, it is prolonged over time and transforms into chronic inflammation. In this microenvironment, cancer cells (including CSCs) can hijack DCs, thereby preventing the presentation of tumor antigens, and in addition, they can recruit a variety of immunosuppressive cells (e.g., MDSCs, Tregs, M2-TAMs, and N2-TANs) by producing cytokines, chemokines, and inflammatory mediators. The resulting environment is rich in pro-angiogenic and pro-tumorigenic factors and prevents innate immunity and the T cell response from exerting anti-tumor effects [111,112].

The degree of inflammation may vary depending on the type of tumor. Some tumor types are specifically inflammatory, such as liver, gastric, colorectal, or breast cancer [66,113].

CSC niches have been identified in a number of human cancer types, such as esophageal [114], gastric [115], colorectal [116], liver [117], pancreatic [118], breast [119], ovarian [120], prostate [121], renal [122], brain [123], head and neck [124], lung [125], or melanoma [126]. Numerous studies have examined the similarities and variations between the habitats of various malignancies, as well as the effect of cancer-specific microenvironments on the establishment and growth of CSCs [127,128,129,130,131,132,133]. In order to gain a better understanding of the interaction between tumors and CSC niches, as well as the role of inflammatory milieu in the maintenance of various CSCs, we will now examine some of the most significant, highly inflammatory cancers.

In the microenvironment of the liver, CSCs promote pro-tumor TME formation in several ways, such as the production of the tissue inhibitor of metalloproteinase 1 (TIMP1) or the activation of the hepatocyte-derived growth factor/hepatocyte-derived growth factor receptor (HGF/HGFR) system by the hypoxia-induced activation of HIF1. Kupffer cells and neutrophils enhance tumor necrosis factor (TNF)α, IL1, and MMP9 production as well [134]. The production of growth factors (e.g., epidermal growth factor receptor /EGFR/, VEGF, PDGF, and stromal cell-derived factor 1 /SDF1/), TNF, and other angiogenic factors also promotes the growth and survival of CSCs and their resistance to radiotherapy and chemotherapy [135,136,137,138]. A number of surface markers are known for CSCs in liver cancer (e.g., CD133, CD90, CD24, CD13, epithelial cell adhesion molecule /EpCAM/, aldehyde dehydrogenase /ALDH/, and hepatic progenitor cell marker OV-6). The expression of stem cell markers confers different properties to CSCs. In CD90^+^ cells, genes associated with inflammation and drug resistance are upregulated, whereas CD133^+^ cells are resistant to apoptosis and radiotherapy through activation of the Ak strain transforming/Protein kinase B (Akt/PKB) pathway [139,140].

Gastric CSCs have been identified in several cell lineages and are characterized by several stem cell markers (e.g., CD44, ALDH, CD54, CD24, CD71, CD326, CD49f, CD54, CD90, CD133, SRY-box transcription factor 2 /SOX2/, octamer-binding transcription factor 4 /OCT4/, NANOG) [141,142,143]. Infection with Helicobacter pylori has been shown to favor the development of gastric cancer CSCs in animal models [141,142,143]. The chronic inflammation caused by such an infection is also helpful for the development and maintenance of the CSC niche [141,142,143].

CD44, CD133, CD166, leucine-rich repeat-containing G-protein coupled receptor 5 (Lgr5), ALDH1, EpCAM, and other more general markers such as NANOG, SOX2, OCT4, CD51, CD24, CD26, and CD29 are used to identify colorectal CSCs [144]. The expression of CD133, OCT4, and NANOG in colitis-associated cancers (CACs) are significantly lower than in sporadic CRC [144]. Additionally, although recent research identifies the Lgr5^+^ stem cells as the possible cells of origin for the formation of mice adenoma and human CRC [145,146,147], the proportion of Lgr5^+^ CSCs in CAC is one third less than in CRC [148]. This confirms that the molecular pathogenesis of CAC is distinct from that of sporadic CRCs, as genomic alterations appear to be directly connected to the effects of chronic inflammation and repetitive mucosal injury in inflammatory bowel diseases [149]. Using an azoxymethane/dextran sodium sulfate (AOM-DSS) mouse model of CAC, it was demonstrated that DSS isolates colonic epithelial stem cells from both the stem cell niche and the Wnt signaling-supporting basal lamina. In doing so, the DSS stops the stem cell program. Within ex vivo circumstances, niche damage caused by a progressively increasing dose of DSS promoted the formation of Wnt-independent dysplastic organoids. These organoids contain tenfold more Lgr5^+^ colonic epithelial stem cells and have orthologous Wnt mutations to human CRC driver mutations. These suggest that CRC is formed by the niche injury-induced outgrowth of normally suppressed mutant stem cells [150]. Deletion of the aryl hydrocarbon receptor (AhR) increases the number of Lgr5^+^ stem cells and enhances their organoid-initiating capacity. In a colorectal inflammatory tumor model, AhR knockdown in intestinal epithelial cells increases basal stem cell and crypt injury-induced cell proliferation by upregulating forkhead box M1 (FoxM1) signaling and promotes colitis-associated carcinogenesis [151].

Different populations of breast cancer CSCs can give rise to different tumor cell lines. Breast CSCs that are CD44^+^/CD24^−^ are known to be associated with intra-tumoral inflammation and tumor-infiltrating CD4^+^ T cells [152]. However, the genotype of the new cells may not resemble the genetic profile of the original CSCs, which may indicate the development of mutations [153]. The negative feedback balance between the tumor suppressor breast cancer gene 1 (BRCA1) and the transcription factor SNAI2 gene (Slug) is a key element to maintaining normal tumor growth and determining TME’s stem cell concentration [154,155]. Overexpression of the SOX family promotes EMT and upregulates the expression of the enhancer of zeste homolog 2 (EZH2), which plays an important role in the histone methylation of several genes and can activate the Raf-1 proto-oncogene, serine/threonine kinase (Raf1)/β-catenin pathway [156,157].

## 5. Emergence of CSC Phenotype without TME

Interestingly, the activation of the TLR9 inflammatory signaling pathway, which is part of the innate immune system, can result in the CSC phenotype without the presence of TME. Regarding cell-free DNA (cfDNA), it has been shown that the structure and origin of cfDNA influence its biological effects on cancer cells [158]. Using an in vitro cellular model that lacked both the TME and the immune system of the tumor-bearing host, we investigated the pathobiological effects of self-DNA administration in HT29 colon cancer cells. We provided evidence [159] for a close existing interplay between TLR9 signaling and the autophagy response, which had significant effects on tumor cell survival in HT29 cells treated with intact or modified self-DNA. Interestingly, we also found colonosphere formation with a strong cytoplasmatic CD133 immunoreactivity in artificially hypermethylated DNA-treated HT29 cells. We further discovered [160] that the combined use of tumorous self-DNA and IGF1R inhibition displays anti-proliferative properties that can be suppressed by inhibiting TLR9 signaling. Autophagy induced by self-DNA and IGF1R inhibitor also resulted in the survival of CD133-positive HT29 stem-like cancer cells, which may play a role in the CRC recurrence. Since HT29 cancer cells are wildtype regarding Kirsten rat sarcoma virus (K-Ras) mutation, it cannot be ruled out that this observed phenomenon is partly mediated by the RAS/extracellular-signal-regulated kinase (ERK) and phosphoinositide 3-kinase (PI3K)/Akt pathways, with a close connection to the pro-inflammatory factors like IL17, IL22, and IL23 [161]. However, it should not be overlooked that while HT29 cells are able to express CD133, there are colon cancer cells (e.g., HCT15, LS180, SW480, DLD1, and COLO205) that do not express CD133 [162].

## 6. Utilization of the Inflammatory Process in Cancer Therapy

Theoretically, a number of new ways to boost the immune response against tumors seem to be able to control inflammation caused by cancer.

Local inflammation generated by irradiation or oncolytic viruses can stimulate an anti-cancer innate immune response by activating nucleic acid receptors (TLR9, cyclic GMP–AMP synthase /cGAS/-stimulator of interferon genes /STING/, or RIG-I like receptor /RLR/), followed by a type I IFN response [158]. The PI3K/Akt/mammalian target of rapamycin (mTOR) pathway is one of the major signaling pathways in CSCs involved in stemness maintenance, proliferation, differentiation, EMT, migration, and autophagy. Therefore, inhibiting the PI3K/Akt/mTOR pathway may also be a promising targeted cancer treatment method [163].

Restricting the infiltration and function of immunosuppressive cells (e.g., MDSCs, Tregs, M2-TAMs, and N2-TANs) may restore immune surveillance by blocking inflammatory pathways [111]. Immunotherapeutic strategies such as immune checkpoint blockage, monoclonal antibodies, vaccination, CD8^+^ T cell treatment, and activation of innate immune responses such as NK cells, cytokine-induced killer cells, can be used to target CSCs [164,165]. Loss of cancer antigen expression, activation of oncolytic pathways, and promotion of an immunosuppressive milieu and (epi)genetic modifications that diminish their identification by the immune system are just a few of the ways that CSCs have developed to evade a potential attack from the immune system. These immunotherapeutic techniques have the potential to increase CSCs’ sensitivity to chemotherapy and/or radiotherapy. Immune checkpoint blockage strategies, for instance, can inhibit the immunosuppressive activity of CSCs and other immunosuppressive cells inside the TME. CSCs and cancer cells generate PD-L1, whereas Tregs express its receptor PD-1 [107,166,167]. CSCs also distribute PD-1 to their respective specialization [165]. PD-L1 could lead to the depletion and malfunction of effector T cells [168] and prevent CSCs from evading the immune system. Importance is placed on employing immunotherapeutic strategies targeting specific CSC markers and antigens that are preferentially expressed by the cells [165].

Recent studies on the epigenetic regulation of CSCs by histone lysine methyltransferase and histone demethylase inhibitors have received significant attention [169,170,171]. Moreover, because signaling pathways play key roles in stimulating the proliferation of CSCs, maintaining the phenotype of CSCs, and in embryonic development, therapeutic strategies targeting these pathways have been discovered. Included among these signaling pathways are NF-kB, Janus kinase (JAK)-STAT, and TGFβ/suppressor of mothers against decapentaplegic (Smad). Specifically, addressing epigenetic alterations in signaling networks has emerged as a potential tumor therapy research approach. Tocilizumab, for instance, blocks IL6/STAT3 signaling and reduces the cancer/inflammation epigenetic IL6/STAT3/NF-kB positive feedback loop, which is of immense therapeutic utility for patients with resistant triple-negative breast cancer [172]. In addition, activation of the NF-kB pathway in pancreatic cancer stem cells is dependent on methylation of the downstream regulatory gene SOX9, and DNA methyltransferase inhibitors may represent a novel therapeutic option for pancreatic cancer treatment [173].

Besides targeting CAF-derived components, depleting pro-tumor CAFs or transforming them into dormant or anti-tumor cells are all potential anti-cancer therapeutic strategies as well [174,175,176,177,178,179]. The capacity of CAFs to confer stemness to cancer cells renders this treatment possibility intriguing. Compared to epithelial cancer cells, immunological cells, and endothelial cells, CAFs are more positively connected with gene sets associated with poor prognosis, providing support for targeting CAFs as a viable therapeutic route. However, the variety of CAFs needs the identification of more specific markers, as there are CAFs that inhibit tumor growth. Intriguingly, the tumor devoid of myofibroblasts displayed improved spheroid formation, indicating a higher proportion of CSCs. Indeed, the identification of CAF subtypes demonstrates that they promote or inhibit tumor progression in a tissue-dependent way, supporting the necessity for additional research into CAF-specific indicators [180]. Reeducating pro-tumor CAFs into a state of quiescence or even anti-tumor CAFs is an attractive concept. Given that vitamins (e.g., all-trans retinoic acid or the vitamin D metabolite 1α, 25-dihydroxyvitamin D3) are essential for healthy tissue and their toxicity is relatively lower compared to chemotherapy, reusing vitamin analogs to reconfigure stimulated fibroblasts into a quiescent state may be a clinically viable therapeutic strategy [177,178]. Reprogramming the fibroblasts using growth factors, as evidenced by the flexibility of CAFs, is another technique for rewiring the fibroblast population. TGFβ inhibits an IL1-induced phenotype and drives the fibroblast to acquire a myofibroblastic phenotype with less carcinogenesis, including reduced expression of markers supporting cancer stemness, such as IL6 and CXCL12 [179]. This justifies the option to convert tumor-promoting fibroblasts into tumor-restraining fibroblasts. To account for the cancer cell’s potential to activate tumor-promoting CAFs, inhibitors may be utilized to circumvent cancer cell-mediated CAF activation.

Anti-VEGF resistance causes unsuccessful cancer treatment and recurrence. (Epi)genetic changes cause acquired resistance in cancer cells [181]. Tumor ECs have epigenetic changes that contribute to anti-angiogenic treatment resistance. Anti-resistance therapy may include many anti-angiogenic substances or anti-angiogenic drugs combined with other treatments. Intussusceptive microvascular formation, vasculogenic mimicry, and vascular co-option are anti-angiogenic treatment resistance mechanisms. Angiogenesis and immune cells interact, which is why anti-angiogenic and immunological checkpoint drugs work so well together. Pan-omics profiling improves clinical outcomes and fights anti-angiogenesis drug resistance [181].

It is necessary to develop reliable tests for measuring stem cell function in human specimens. Identification of CSCs and tracking of anti-CSC treatment effectiveness in clinical samples rely mostly on surface markers at present. Due to the constraints of marker-based selection and the flexibility of the CSC state, it is crucial to optimize functional assays as a validation of self-renewal to eradicate all subclones of CSC [182]. Window-of-opportunity trials, in which surgery follows targeted therapy, provide an opportunity for comprehensive evaluation of the therapy response, including comparisons of the CSC rate, stemness indicators, genetic signatures, and functional assays (e.g., xenotransplantation or surrogate in vitro assays) with the diagnostic sample. Specifically, the determination of CSC genetic signatures can aid in patient stratification (risk assessment), identification of therapy response (surrogate markers), and/or differential diagnosis (identifying who is most likely to be responsive to which medications). Before biomarkers can be used regularly in clinical practice, they must be validated through clinical research [182,183]. This research must take into account a number of important factors, such as scientific reasoning, clinical trial design, marker evaluation methods, cost, and feasibility.

Even if the entire remaining combination of immune-evasive strategies is successfully targeted by experimental therapies, novel, as-yet-unknown mechanisms are likely to emerge to thwart therapeutic efforts; consequently, a continually changing, thorough knowledge of TME biology is essential for preparing for the future. New discoveries in basic biology will unquestionably lead to ways of surpassing tumor development, which seeks to elude pharmacologic and biologic therapy, as well as more effective strategies for eradicating CSCs. Future research should focus on integrating these therapies into combination immunotherapy regimens and limiting the effects of these approaches to the site of action in order to minimize systemic pro-inflammatory effects. Also, in the field of gene therapy, monoclonal antibodies against cytokines or cell-based medicines may work well with small molecule-based targeted therapies to kill cancer cells (including CSCs) for a long time. More precise and personalized approaches need to be tested in well-designed clinical trials. However, this is challenging because the relationship between CSCs and inflammatory TME is extremely complex; it is therefore almost impossible to identify a single or small number of therapeutic targets whose manipulation will exclusively result in a beneficial therapeutic effect. Hence, it is obvious that more research is required to improve cancer treatment strategies based on targeting tumor-promoting inflammation.

## 7. Conclusions

CSCs are capable of altering their own properties in a variety of ways to preserve their stem cell phenotype, resist different therapies, and evade the immune system’s anti-tumor attack. Through immune escape mechanisms, they are not only able to hide themselves from the immune system but also to influence the anti-tumor immune elimination mechanisms in a way that is favorable to them. By manipulating their own capabilities, CSCs have the potential to develop entirely novel anti-cancer treatments and methods to prevent disease recurrence.

It is clear that there is an intense and complex multi-level relationship between the TME and CSCs. CSCs are able to develop an inflammatory niche that allows them to persist and divide on their own. They maintain an intense relationship with the cellular elements of the TME, reprogramming them into cells for the survival and proliferation of CSCs. In turn, the reprogrammed TME cells enhance the survival and proliferation of CSCs and thereby facilitate their own survival and function. If we can understand all the elements of the cross-talk between CSCs and TMEs, the development of a number of novel and targeted anti-cancer therapies will become possible.

Tumors can also be distinguished by their characteristic inflammatory infiltration. In the case of tumors with a highly inflammatory character, stemness markers play a major role, as they not only affect the stem cell phenotype of CSCs but also play a major role in the maintenance and regulation of pro-tumor inflammation. A better understanding of CSC markers and their relationship to inflammation could also serve as a starting point for potential anti-cancer therapies.

It is also a very important observation that the CSC phenotype can be expressed without TME simply by triggering certain signaling pathways involved in inflammation. This will allow the development of potential anti-CSC phenotype treatment strategies in vitro.

Many efforts have been made over the past few decades to uncover the mechanisms through which inflammation promotes carcinogenesis. Few of these studies attempt to propose a theoretical hypothesis and advance our understanding of the underlying laws governing inflammation-induced carcinogenesis, whereas the majority of these investigations give segmental and fragmentary evidence. The theory of “Cancer Evo-Dev” began by reviewing research findings on hepatitis B-induced hepatocarcinogenesis, then moved on to other inflammation-related carcinogenesis [184]. This new idea not only aids in the comprehension of the mechanisms by which inflammation promotes the development of malignancies, but it also lays the groundwork for the creation of targeted cancer prevention and treatment. However, it should not be forgotten that modulation of the inflammatory immune response can be a double-edged weapon that, under inappropriate conditions, can cause CSCs to survive, divide, and spread, and thus cause cancer progression or recurrence. In view of these findings, we believe that further experimental investigation of the relationship between the inflammatory microenvironment and cancer stem cells is warranted.

## Figures and Tables

**Table 1:** In the most commonly investigated cancers, CSC-related markers have been revealed. It is important to note that several of these markers may also be expressed by healthy stem cells and normal tissue cells (for more information on this topic, see references [3,4,5,6]). EpCAM: Epithelial cell adhesion molecule; CXCR4: C-X-C chemokine receptor type 4; Lgr5: Leucine-rich repeat-containing G-protein coupled receptor 5; ProC-R: Protein C receptor; LINGO2: Leucine rich repeat and Ig domain containing 2; CLL-1: C-type lectin-like molecule-1; TIM3: T-cell immunoglobulin and mucin domain 3; IL1RAP: Interleukin 1 receptor accessory protein; OV-6: hepatic progenitor cell marker; ESA: epithelial surface antigen; DCLK1: Doublecortin-like kinase 1; ABCB1: ATP Binding Cassette Subfamily B Member 1; CHL1: close homolog of L1; TACSTD1: tumor-associated calcium signal transducer-1; ALDH: Aldehyde dehydrogenase; Nanog: Nanog Homeobox; Oct-3/4: Octamer-binding transcription factor 3/4; BMI-1: B-cell-specific Moloney murine leukemia virus integration site-1; SOX2: SRY-Box Transcription Factor 2; Letm1: Leucine zipper-EF-hand containing transmembrane protein 1; FOXO: Forkhead transcription factor family O; Sall4: Spalt-Like Transcription Factor 4; AFP: alpha-fetoprotein; KLF4: Kruppel-like factor 4; NES: neuroepithelial stem cell protein; TGM2: transglutaminase 2 gene.

		Cancer Stem Cells										
		Lung	Breast	Gastric	Colorectal	Liver	Pancreas	Melanoma	Glioblastoma	Prostate	Acute Myeloid Leukemia	Chronic Myeloid Leukemia
Surface CD markers	CD15								+			
	CD24		+	+	+	+	+					
	CD25								+			+
	CD26											+
	CD29		+									
	CD33										+	+
	CD36											+
	CD44 (and variants)	+	+	+	+	+	+		+	+		
	CD49f		+									
	CD61		+									
	CD70		+									
	CD87	+										
	CD90	+	+	+		+			+			
	CD117									+		+
	CD123										+	+
	CD133	+	+	+	+	+	+	+	+	+		
	CD166									+		
	CD271							+				
	CD274					+						
Other surface markers	EpCAM	+	+	+	+	+				+		
	CXCR4		+	+			+					
	Lgr5		+	+	+							
	ProC-R		+									
	LINGO2			+								
	CLL-1										+	
	TIM3										+	
	IL1RAP											+
	cell surface vimentin				+							
	OV-6					+						
	ESA						+					
	DCLK1						+					
	ABCB1						+					
	CHL1								+			
	TACSTD2									+		
Intracellular markers	ALDH	+	+	+	+			+	+	+	+	
	Nanog	+	+	+	+	+		+	+		+	
	Oct-3/4	+	+	+	+	+	+	+			+	
	BMI-1		+									
	SOX2		+	+	+	+	+	+	+		+	
	Letm1			+	+							
	Musashi2			+								
	FOXO											+
	Sall4				+				+			
	AFP					+						
	KLF4								+			
	NES								+			
	TGM2									+		

## Data Availability

Not applicable.
